# Effects of 36-hour recovery on marksmanship and hormone concentrations during strenuous winter military survival training

**DOI:** 10.1186/s13102-023-00711-6

**Published:** 2023-08-18

**Authors:** Tommi Ojanen, Kai Pihlainen, Jussi Yli-Renko, Jani P. Vaara, Tarja Nykänen, Risto Heikkinen, Heikki Kyröläinen

**Affiliations:** 1 Finnish Defence Research Agency, Human Performance Division, Finnish Defence Forces, Tuusula, Finland; 2 Defence Command, Training Division, Finnish Defence Forces, Helsinki, Finland; 3 Department of Leadership and Military Pedagogy, National Defence University, Finnish Defence Forces, Helsinki, Finland; 4 Army Academy, Finnish Defence Forces, Lappeenranta, Finland; 5 Statistical Analysis Services, Analyysitoimisto Statisti Oy, Jyväskylä, Finland; 6 Faculty of Sport and Health Sciences, University of Jyväskylä, Jyväskylä, Finland

**Keywords:** SERE, Soldier, Endocrinology, Arctic

## Abstract

**Objectives:**

Survival training can provide a unique setting for scientific examination of human stress responses and physical performance in a realistic operational military context. The aim of the present study was to observe effects of a 36-h recovery period on serum hormone concentrations, salivary cortisol, and marksmanship during 10-day winter military survival training in north of the Arctic Circle.

**Design and methods:**

Sixty-eight male soldiers were randomly divided into two groups; EXP (*n* = 26) and CON (*n* = 42). While CON performed the whole exercise phase in the field, EXP had 36-h recovery period between days 6 and 8. Several hormones were measured during the study to investigate recovery.

**Results:**

Subjective physical and mental demand as well as catabolic hormone levels increased and anabolic hormones decreased in CON (*p* < 0.05), whereas in EXP, recovery period attenuated negative effects of survival training. Prone shooting performance decreased (87.5 ± 6.5 vs. 76.3 ± 8.8, points out of 100, *p* < 0.05) between days 6 and 8 in CON while EXP was able to maintain shooting performance throughout the study.

**Conclusion:**

A short recovery during a strenuous training can prevent the degradation in psychophysiological state and shooting performance in soldiers, which can be crucial for survival in demanding operational winter environment. In the present study, 36-h rest period during the field training seems to enhance recovery but the duration of the period was inadequate for full recovery from the accumulated operative stress. In conclusion, appropriate recovery periods should be implemented in order to optimize occupational performance during high operative stress.

## Introduction

Environmental extremes, such as cold weather, are stressors in occupations required to operate long periods in ambient temperature [1, 2]. For example, increased fatigue, challenges in thermoregulation, stiffness in the neck and shoulder areas are common sensations among outdoor workers during physical labor in the cold [1], which may increase subjective demand level of any given task. In the military context, soldiers typically face very high levels of operative stress, consisting of sleep and caloric restriction, high levels of physical activity in various environmental extremes and psychological discomfort during survival training [3, 4]. Without proper recovery, readiness of soldiers in the field can be drastically compromised because of hormonal disturbances, negative changes in body composition, and physical performance [3, 5]. Cold climatic and arctic conditions exacerbate the stress during military training [2]. For example, protective clothing increases energy expenditure [6], manual material handling is challenged by decreasing dexterity of fingers and movement in deep snow is impossible without skis, which increases the oxygen consumption during physical activity [7, 8]. Survival training courses (SERE) can provide a unique setting for scientific examination of human stress responses and physical performance in a realistic operational military context [4].

Several biomarkers have been used to measure stress in extreme military settings [4, 9, 10]. Commonly, serum testosterone (TES) and insulin-like growth factor-1 (IGF-1) levels have been shown to decrease during strenuous military training while cortisol (COR) and sex hormone binding globulin (SHBG) have been shown to increase [9–12]. Stress in extreme military environments has been shown to induce acute activation of the sympathetic nervous system (SNS) and the hypothalamic–pituitary–adrenal (HPA) axis, which can be observed as increases in neuroendocrine biomarkers, epinephrine (EPI), norepinephrine (NOR) and cortisol (COR) [4, 13, 14]. Furthermore, sustained physical activity together with negative energy balance and sleep deprivation during military field exercise has been shown to decrease basal levels of anabolic hormones [11, 12, 15]. Most often such changes have been reported for insulin-like growth factor-1 (IGF-1) and testosterone (TES), along with increases in sex hormone binding globulin (SHBG) which additionally limits the levels of bioavailable TES [9–12].

Previous cold environment military studies have concentrated on energy expenditure and energy balance [7, 16, 17], protective clothing [18] and cold weather injuries [19]. Nykänen et al. [17] has recently reported results of the present study population and setting regarding energy balance and changes in body composition. This study showed that winter survival training caused severe energy deficit (more than -4500 kcal/day, as energy expenditure between 5000 to 5500 kcal/day, and energy intake between 500 to 1000 kcal/day) and decreases in body mass and body fat percentage. To date, there are fewer observational studies focused on hormonal profile [20], marksmanship [21] and physical performance [8]. Furthermore, very few studies have reported the effects of recovery or other countermeasures for the decrements in occupational performance during high operative stress [3, 5, 22, 23]. Without countermeasures, recovery back to the baseline levels from short-term (5–10 days) field training varies between the observed variables and depends on duration as well as combination of exposure to operative stressors [3, 9, 22, 23]. According to these studies, recovery of body composition, hormonal status and physical performance may take from few days up more than two weeks.

During World War 2, Finnish army provided a short (3–4 days on average) recovery period close to the front line for highly stressed soldiers. This small-scale experiment, mainly focusing on adequate sleep and provision of food showed that most of the highly stressed soldiers could successfully return to combat duties, while individuals with more severe traumas could be screened for further medical assistance ([24], pp. 116–118). While no scientific data are available from actual combat operations, the aim of the present study was to observe the effects of a 36-h recovery period on hormone concentrations, and marksmanship during 10-day winter military survival training. It was hypothesized that a 36-h recovery period during 10-day strenuous winter survival training would show positive recovery on hormone concentrations and marksmanship.

## Methods

This research was conducted during a regularly scheduled Finnish Army winter survival training lasting 10-days in north of the Arctic Circle. The exercise included practices of different military survival skills like, building temporary shelters, learning how to find and prepare food in winter environment, evasion skill training, and navigating in winter conditions. When the subjects were on the field, they were performing military exercise around the clock and ate, slept if and when it was possible. Sixty-eight male soldiers volunteered to participate in the present study. The participants were randomly divided into two groups; Experimental (EXP) (*n* = 26) and Control (CON) (*n* = 42). CON remained in the field for the whole duration of the study (from 08:00 on day 3 to 08:00 on day 10). EXP had a recovery period in the middle of the field phase from day 6 (18:00) to day 8 (06:00). During this recovery intervention, EXP stayed in an indoor accommodation, where they could rest, eat, drink ad libitum, and take part in relaxation activities [25], such as mindfulness. The duration and content of the field exercise as well as recovery period was planned by the military subject matter experts. The average (± SD) age (yrs.) of the soldiers was 19.7 ± 1.2 (EXP) and 19.6 ± 0.8 (CON), height (cm) 181.1 ± 5.8 (EXP) and 179.4 ± 6.2 (CON), body mass (kg) 78.2 ± 9.6 (EXP) and 74.4 ± 10.7 (CON), and BMI (kg/m^2^) 23.9 ± 2.7 (EXP) and 23.1 ± 2.8 (CON).

**Fig. 1:**
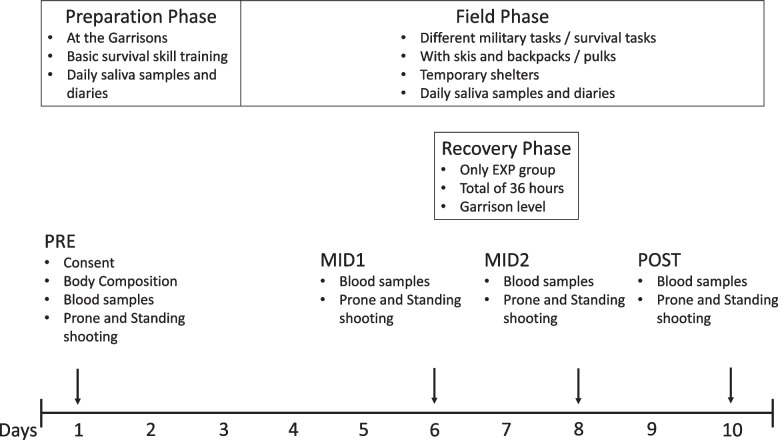
Study design. PRE = pre-measurement, MID1 = before recovery phase, MID2 = after recovery phase

The participants skied on average 19.3 ± 1.7 km/day during the first 3 days of the field phase, and 13.8 ± 1.3 km/day during the remaining days, with the exception of the EXP group which did not ski during their 36-h recovery period. Measurements were conducted four times (PRE (day 1), MID1 (day 6), MID2 (day 8), and POST (day 10)) for studying serum hormone concentrations and marksmanship (Fig. 1). In addition, daily saliva cortisol samples and diaries for rating of daily perceived exertion (RPE 6–20) [26], amount of daily sleep in hours (SL), and NASA task load index (NASA-TLX) [27] were collected. NASA-TLX was assessed independently for mental and physical demands. The diaries were filled every morning and evening by the participants and collected daily along with the saliva samples by the researchers at 8:00 and 20:00. Weather information was collected daily from the data provided by Finnish Meteorological Institute. The temperature varied between -20.9 °C and 5.4 °C (mean; -2.5 °C). The average snow depth was 89 cm (from 78 to 101 cm). This study was performed in line with the principles of the Declaration of Helsinki. The Scientific and Ethical Committee of the Helsinki University Hospital Research (HUS/900/2018) granted an ethical statement and the study was authorized by the Finnish Defence Forces (AO1720). All participants were informed of the experimental design and they provided written informed consent to participate.

Saliva samples were collected daily at 08:00 and 20:00 using Salivette® tubes (Sarstedt, Nümbrecht, Germany). Soldiers were guided by an instructor to keep the swab in the mouth for two minutes. Samples were then collected, centrifuged, frozen and analyzed afterwards for saliva cortisol (sCOR) with Immulite 2000 XPi (Siemens Helthcare, UK) using chemiluminescent enzyme immunoassay kits (interassay difference 13%).

Venous blood samples (VenoSafe®, Terumo Europe, Leuven, Belgium) were drawn four times during the study after an overnight fast from the antecubital vein between 06:00 and 07:00. The samples were centrifuged (Megafire 1.0 R Heraeus, DJB Lab Care, Germany) after 30 min at 2000 g for 10 min, aliquoted, frozen, and transported to the laboratory (University of Jyväskylä) for analysis. Serum hormone concentrations of TES, COR, SHBG, IGF-1, and dehydroepiandrosterone (DHEA-S) were analyzed (Siemens Immulite 2000 XPI, Siemens Healthcare, USA). Plasma catecholamines, EPI, and NOR were determined via High Performance Liquid Chromatography (HPLC) (Quest Diagnostics, USA), and creatine kinase (CK) activity with Konelab 20 XTI (Thermo Scientific, Finland). All the measures were performed in duplicate with intra- and interassay differences typically under 5 and 10%, respectively.

The shooting accuracy test was performed indoors to ensure standardized conditions from prone and standing positions with a replica Army assault rifle (RK95, Finland) system (Eko-Aims Ltd, Finland). The participants were familiar with practices in both shooting positions from their preceding military service as well as handling the weapon since identical assault rifle is provided to all conscripts in the beginning of their military service. Ten shots were fired and the results for each participant were electronically determined with an accuracy of 0.1 points (range 0–10 points/shot), with the best possible total score of 100 points when all shots hit the center of the target.

Statistical analysis was conducted in R (R Core Team, 2020). Data are presented as means with standard deviation (± SD) and statistical significance was set at *p* < 0.05. A linear mixed effect model was used to maximize observations at each time point. Pairwise comparisons were performed using Tukey’s test and logarithmic transformations were done when the distribution was positively skewed. Non-parametric Mann–Whitney U-tests were used to verify the main conclusions of linear mixed effect model when residuals were not normally distributed. Pearson correlations were calculated combining groups EXP and CON to estimate associations between the performance tests.

## Results

**Fig. 2:**
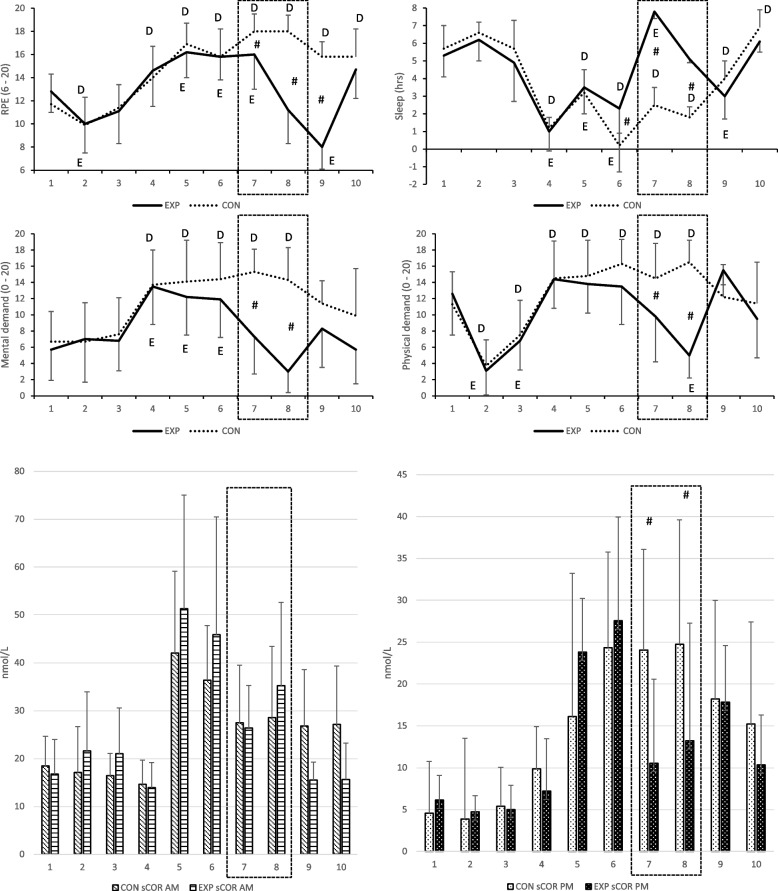
Daily RPE (rate of perceived exertion), sleep, mental and physical demands, and saliva cortisol (sCOR) AM and PM values during SERE in both EXP and CON groups. D and E = *p* < 0.05 compared to day 1 value, # = difference between groups *p* < 0.05. Experimental period is highlighted with dashed line

Subjective physical and mental demand levels (Fig. 2) reached peak values by days 6–7 in CON, whereas the values rapidly improved by day 8 in EXP. Physical demand levels (NASA-TLX) increased between days 6 and 8 by 2% in CON, whereas for EXP, the recovery period decreased the physical demand levels by 48%. Even higher changes were observed in both groups regarding mental demand levels: which increased in CON by 23% between days 6 and 8 while the respective change in physical demand levels of EXP was a decrease of 69%.

No changes were observed in standing shooting accuracy in either group (Table 1). Prone shooting, however, decreased significantly between MID1 and MID2 (87.5 ± 6.5 vs. 76.3 ± 8.8, points out of 100, *p* < 0.05) in CON while EXP was able to maintain shooting performance throughout the study.

**Table 1:** Shooting scores (prone and standing) of the EXP and CON groups during the study. Theoretical maximum score is 100. A = *p* < 0.05 compared to PRE, B = *p* < 0.05 compared to MID1, C = *p* < 0.05 compared to POST, # = *p* < 0.05 between the groups

		PRE	MID1	MID2	POST
Standing (points)	EXP	68.8 ± 11.4	67.6 ± 11.3	68.5 ± 11.1	67.0 ± 12.5
	CON	62.0 ± 10.3 #	63.0 ± 11.2	64.2 ± 11.4	63.6 ± 10.4
Prone (points)	EXP	93.3 ± 5.6	92.7 ± 3.2	91.0 ± 5.5	91.8 ± 7.4
	CON	85.9 ± 10.4 #	87.5 ± 6.7	76.3 ± 9.0 A,B,C	85.6 ± 7.4

**Fig. 3:**
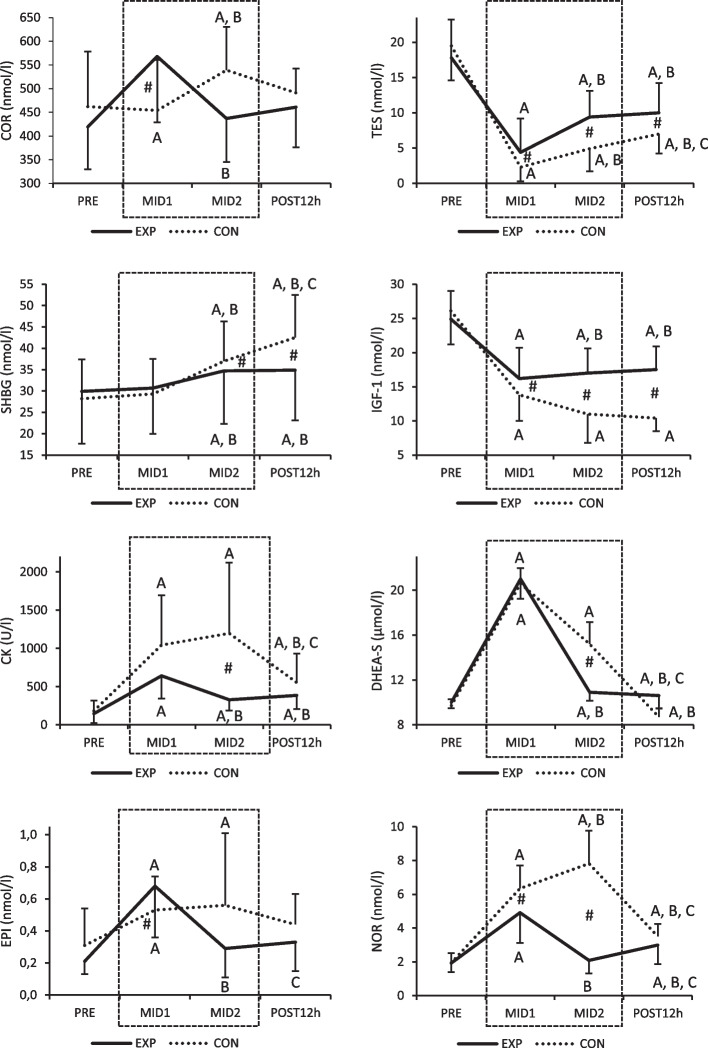
Serum biomarker profiles during the study. CO*R* = cortisol; TES = testosterone; SHBG = sex hormone-binding globulin; IGF-1 = insulin-like growth factor 1; CK = creatine kinase; DHEA-S = dehydroepiandrosterone sulfate; EPI = epinephrine; NO*R* = norepinephrine. A = *p* < 0.05 compared to PRE, B = *p* < 0.05 compared to MID1, C = *p* < 0.05 compared to MID2, # = difference between groups *p* < 0.05. Experimental period is highlighted with dashed line

Anabolic hormones followed identical decreasing patterns from the baseline in both groups, but recovery period of EXP increased IGF-1 levels from MID1 significantly, whereas in CON, the respective levels remained decreased. In EXP, COR levels increased at MID1, while similar increase was observed in CON later at MID2. On the contrary, at that time point COR levels returned back to baseline level in EXP during the recovery period. Catecholamines increased significantly in both groups by day 6 but thereafter, recovery period returned EPI and NOR levels of EXP to the baseline, whereas in CON, the respective levels remained elevated until day 8. By the end of the study, NOR increased again in EXP and when compared to the baseline, remained elevated in both groups (Fig. 3).

Changes in saliva cortisol (sCOR) levels followed an increasing trend (Fig. 2). Furthermore, as saliva samples were collected twice a day, diurnal variation was observed to almost diminish in both groups by day 6 (EXP 45.9 ± 24.6 nmol/l (AM), 27.5 ± 18.3 nmol/l (PM); CON 36.4 ± 11.4 nmol/l (AM), 24.3 ± 12.4 nmol/l (PM)). Recovery period of EXP returned variation towards normal reference levels (day 7; 26.4 ± 11.3 nmol/l (AM), 10.5 ± 4.0 nmol/l (PM) and day 8; 35.2 ± 13.9 nmol/l (AM), 13.2 ± 5.4 nmol/l (PM)), while in CON no variation was observed between morning and evening saliva cortisol samples during days 7 and 8 (day 7; 27.5 ± 12.0 nmol/l (AM), 24.0 ± 12.4 nmol/l (PM) and day 8; 28.6 ± 14.9 nmol/l (AM), 24.7 ± 14.0 nmol/l (PM)).

The change in subjective physical demand level between days 6 and 8 correlated with the respective changes in TES (*r* = -0.57, *p* < 0.01), COR (*r* = 0.45, *p* < 0.05), IGF-1 (*r* = -0.55, *p* < 0.01), NOR (*r* = 0.62, *p* < 0.01) and CK (*r* = -0.59, *p* < 0.01). Similarly, change in mental demand level between days 6 and 8 correlated with change in IGF-1 (*r* = -0.65, *p* < 0.01) and CK (*r* = 0.57, *p* < 0.01), and changes in RPE correlated with changes in IGF-1 (*r* = -0.75, *p* < 0.001), NOR (*r* = 0.59, *p* < 0.01), and CK (0.49, *p* < 0.05) (Table 2).

**Table 2:** Correlations between days 6 and 8 from the changes in serum hormonal markers and the changes in subjective feelings on mental and physical stress

	Mental stress		Physical stress		RPE	
	*r*	*p*	*r*	*p*	*r*	*p*
TES	-0,26	0,274	*-0,57*	0,007*	-0,13	0,638
DHEA-S	0,15	0,564	0,34	0,147	0,30	0,207
COR	0,40	0,088	*0,45*	0,048*	0,39	0,088
IGF-1	*-0,65*	0,002*	*-0,55*	0,009*	*-0,75*	0,001*
SHBG	0,11	0,669	0,31	0,196	0,41	0,075
NOR	0,39	0,089	*0,62*	0,003*	*0,59*	0,005*
CK	*0,57*	0,007*	*0,59*	0,005*	*0,49*	0,027*
EPI	0,36	0,107	0,23	0,339	0,29	0,211

Significant changes marked in italic

*TES* Testosterone, *DHEA-S* Dehydroepiandrosterone, *COR* Cortisol, *IGF-1* Insulin-like growth factor-1, *SHBG* Sex hormone binding globulin, *NOR* Norepinephrine, *CK* Creatine kinase, *EPI* Epinephrine, *RPE* Rating perceived exertion

## Discussion

The 36-h recovery during strenuous winter military survival training attenuated many of the negative effects often accumulated by sustained operative stress. Positive effects included psychophysiological recovery and maintenance of prone shooting performance. Regarding hormonal changes, recovery period attenuated the increases in catabolic hormones and facilitated increases in anabolic hormones when compared to CON. Correlations between subjective stress and catabolic hormone levels reflected psychophysiological relationships in homeostatic regulation. A short recovery during strenuous training can prevent the decrease in occupational performance in soldiers, namely marksmanship in the present study, which can be crucial for survival in demanding operational winter environment. These results underline that after intense military field training critical changes can be expected, along with decrements in hormonal profile with a slow recovery process, to last many days, depending on the training load experienced during the field phase.

Marksmanship, measured as shooting accuracy performance, was maintained in EXP throughout the study while significant decreases were observed in CON during the MID1 and MID2 measurement points, especially in prone shooting. Several factors may be related to this finding. Firstly, the recovery period enabled the participants of EXP to compensate the experienced sleep deprivation and enhance concentration and vigilance for maintaining shooting performance. Anecdotally, we observed that some individuals had trouble in staying awake during the prone shooting and expectedly, their shooting results may have been affected by sleep deprivation. Although we did not measure cognitive performance in the present study, it is possible that the two shooting tests induced different arousal responses, which may, together with sleep deprivation, explain changes in results between EXP and CON. A recent study investigated the effect of sleep deprivation and high military operational stress on attentive abilities, response inhibition, and arousal [28]. The study showed that while the overnight training without sleep led to an impairment in upregulating arousal and attention, soldiers were able to maintain sustained attention and response inhibition in a more stimulating and engaging task. Thus, it is possible that while standing shooting performance was more engaging task, requiring maintenance of stance and upper body muscle activity to hold the weapon for both groups, prone position enabled impairment in upregulating arousal and attention in sleep-deprived EXP, explaining weakening of the results as compared to CON. Secondly, drastic continued energy deficit and higher physical demands of CON may have had more detrimental effects on fine motor performance, such as shooting performance [17, 29, 30].

Strenuous winter survival training disrupted endocrine homeostasis in the present study, reinforcing previous findings. Increased levels of COR, SHBG, EPI, NOR have been reported with decreased levels of TES, IGF-1 after 5–10 days of military field training in several studies [4, 9, 10, 12, 22, 23]. Regarding recovery, Szivak et al. [4] reported that after a 10-day SERE course, 24 h seemed to be enough for recovery in EPI, but not TES, COR, or NOR. Kyröläinen et al. [11] and Vikmoen et al. [31] observed full recovery for COR within 72 h after strenuous military training. Depending on the stress exposure time and biomarkers at hand, recovery after strenuous military field training may also take longer [4, 9, 10, 32]. One week of recovery seems to be enough for TES, IGF-1, SHBG, and CK, but not for COR to recover, according to Hamarsland et al. [9]. In the present study, 36-h recovery in between survival training was adequate to recover all neuroendocrine biomarkers (EPI, NOR, COR) to the baseline concentration levels, although their level increased again when EXP continued their survival training phase. Thus, short recovery period within one week of intensive arctic survival training may be adequate to return homeostasis of the HPA-axis and the sympathoadrenal system. Additionally, 36-h recovery period seemed to return TES towards the baseline, and SHBG and IGF-1 to maintain at the MID 1 level. Regarding CK, DHEA-S, and NOR their values almost recovered to the PRE values, while in CON, the respective values continued to increase. CK increased significantly in CON expressing muscle inflammatory response during survival training. Recovery period in EXP was adequate to partly attenuate catabolic state and by the end of the recovery period, significant difference was observed between the groups. Previous studies have found similar results in a 2-day strenuous mountaineering ski-competition, reporting moderate inverse correlation between CK, COR and energy intake [33].

To the best of our knowledge, this study is among the first ones to report the effects of short recovery period during strenuous winter military field training. Although arctic environment caused challenges in sample collection from all participants and special logistic arrangements had to be organized to conduct the necessary measurements during the field exercise. Nevertheless, the participants were highly motivated to complete the highly physically and mentally strenuous exercise, out of 68 participants who started the study 49 (72%) did complete all measurements, 28 out of 42 (67%) in the CON group and 21 out of 26 (81%) in the EXP group.

In conclusion, combination of physiological and psychological stress during winter military survival training caused severe disturbances in hormonal homeostasis as well as occupational performance in soldiers. Already five days in the field with cumulative effects of caloric restriction, sleep deprivation and highly stressful winter military survival training resulted in significant decreases in anabolic biomarkers, increases in catabolic hormones and diminished diurnal variation in sCOR. One-and-a half day rest period in the middle of the field training seems to enhance recovery but the duration of the period was inadequate for full recovery from the accumulated operative stress. Appropriate recovery periods should be implemented to optimize occupational performance during training. The results of the present study are encouraging, but more research is needed of the optimal duration of short-time recovery and its effects on soldiers performance in a battle situation. Future studies are recommended to test the effects of longer rest periods between the operative stress and further follow-up of recovery should be longer after very demanding field training.

## Data Availability

The data that support the findings of this study are not openly available due to reasons of sensitivity and are available from the corresponding author upon reasonable request.

## Abbreviations

SERE: Survival, Evasion, Resistance, Escape / Survival Training
TES: Testosterone
IGF-1: Insulin-like growth factor-1
COR: Cortisol
SHBG: Sex hormone binding globulin
SNS: Sympathetic nervous system
HPA: Hypothalamic-pituitary-adrenal axis
EPI: Epinephrine
NOR: Norepinephrine
EXP: Experimental group
CON: Control group
PRE: Pre-measurements (day 1)
MID1: Mid-measurements (day 6)
MID2: Mid-measurements (day 8)
POST: Post-measurements (day 10)
RPE: Rating of perceived exertion
SL: Sleep in hours
NASA-TLX: Nasa task load index
sCOR: Saliva cortisol
DHEA-S: Dehydroepiandrosterone
CK: Creatine kinase
